# The first complete mitochondrial genome data of Geoffroy's rousette, *Rousettus amplexicaudatus* originating from Malaysia

**DOI:** 10.1080/23802359.2020.1812449

**Published:** 2020-09-01

**Authors:** Puteri Nur Syahzanani Jahari, Shahfiz Mohd Azman, Kaviarasu Munian, Noor Faradiana M. Fauzi, Mohd Shahir Shamsir, Stine R. Richter, Faezah Mohd Salleh

**Affiliations:** a Department of Biosciences, Faculty of Science, Universiti Teknologi Malaysia, Johor Bahru, Malaysia; b Forest Biodiversity Division, Forest Research Institute Malaysia, Kepong, Malaysia; c Faculty of Applied Sciences and Technology, Universiti Tun Hussein Onn Malaysia, Muar, Malaysia; d Section for Evolutionary Genomics, The GLOBE Institute, University of Copenhagen, Copenhagen, Denmark

**Keywords:** *Rousettus amplexicaudatus*, mitogenome, phylogenetic analysis

## Abstract

The increasing interest in understanding the evolutionary relationship between members of the Pteropodidae family has been greatly aided by genomic data from the Old World fruit bats. Here we present the complete mitogenome of Geoffroy’s rousette, *Rousettus amplexicaudatus* found in Peninsular Malaysia . The mitogenome constructed is 16,511bp in length containing 37 genes; 13 protein-coding genes (PCGs), 22 tRNA genes, two rRNA genes, and a D-loop region. The overall base composition is estimated to be 32.28% for A, 25.64% for T, 14.09% for G and 27.98% for C, indicating a slightly AT rich feature (57.93%). A phylogenetic and BLASTn analysis against other available mitogenomes showed Malaysian *R. amplexicaudatus* matched 98% similarity to the same species in Cambodia and Vietnam. However, it differed considerably (92.53% similarity) with the same species in the Philippines. This suggests flexibility in *Rousettus* sp. with regards to adapting to mesic and dry habitats, ability for long-distance dispersal and remarkably precise lingual echolocation thus supporting its wide-range distribution and colonization. Further taxonomical and mitogenomic comparatives are required in resolving the evolutionary relationship between *Rousettus* spp.

In tropical and subtropical regions of the Old World, fruit bats are an important seed dispersal agents that provide vital ecological services (Chan et al. 2020). Amidst fruit bats, the echolocating bats of the genus *Rousettus* in the Pteropodidae family are widely distributed from Asia to Africa (Hassanin et al. 2020). There are ten *Rousettus* sp. reported worldwide and six of these species occurring within Southeast Asia (Francis 2019). *Rousettus* spp. are selected as the best candidate amongst the Pteropodidae family to study evolutionary relationships as it is the only genus that has the capability of long-distance dispersal and has showed remarkable ecological flexibility in characteristics such as a well-developed echolocation system (Almeida et al. 2016; Stribna et al. 2019; Hassanin et al. 2020). However, lack of genomic data of Asian *Rousettus* sp. could hinder the extensive study of the genus. In this study, we sequenced and provided the whole mitochondrial genome of *R. amplexicaudatus*, which is the first mitogenome of *Rousettus* species from Malaysia.

Muscle tissue was obtained from Gading Forest Reserve, Selangor, Malaysia (Latitude: 3° 21′ 0″ N Longitude: 101° 15′ 0″ E) (Munian et al. 2020). The specimen voucher number MZF1072 was deposited in the Zoological Collection of Forest Research Institute Malaysia (FRIM). The methodologies used for DNA isolation, library construction, read assembly, and gene annotation are described in (Mohd Salleh et al. 2017; Jahari et al. 2020). The mitogenome of *R. amplexicaudatus* from this study (MT259592) is a circular molecule with 16,511 bp in length. Similar to other volant mammals, it contained 13 protein-coding genes (PCGs), 22 transfer RNA genes, 2 ribosomal RNA genes and 1 D-loop region (Yoon et al. 2016; Hassanin et al. 2020). The overall base composition of *R. amplexicaudatus* is estimated to be 32.28% for A, 25.64% for T, 14.09% for G and 27.98% for C, indicating a slightly AT rich feature (57.93%). The total length of the protein-coding gene sequences (PCGs) is 11,405bp. The total length of the 22 tRNA genes is 1509 bp, ranging from 57 bp (tRNASer) to 74 bp (tRNALeu). The 12S rRNA gene length is 969 bp and the 16S rRNA gene length is 1571 bp, and are located between the tRNAPhe and tRNALeu, and are separated by the tRNAVal gene. The control region is located between tRNAPro and tRNAPhe genes. The genes mostly located on the heavy (H) strand except for NAD6 and eight tRNAs genes (tRNAGln, tRNAAla, tRNAAsn, tRNACys, tRNATyr, tRNASer, tRNAGlu, tRNAPro), which were found to be located on the lower (L) strand.

The Malaysian *R. amplexicaudatus* mitogenome shows 98% similarity to the same species from Cambodia (MN816352.1) and Vietnam (MN816353.1) (Hassanin et al. 2020) available in Genbank. Interestingly, it diverged by nearly 8% (92.53% match) for the same species originating from the Philippines (MN125184.1) (Mendoza & Fontanilla 2019). In Figure 1, the phylogenetic analysis of this relationship of the *Rousettus* sp. mitogenomes is depicted. The phylogenetic tree generated a monophyletic clade of *R. amplexicaudatus* from Malaysia, Cambodia and Vietnam meanwhile; Philippines *R. amplexicaudatus* formed another branch. Unlike other fruit bats, *Rousettus* sp. are reported to be more adaptive to certain biogeographic barriers. Their flexibility to adapting in mesic and dry habitats, ability for long-distance dispersal and remarkably precise lingual echolocation support the species wide-range distribution and colonization (Happold & Happold 2013; Stribna et al. 2019; Hassanin et al. 2020). Therefore, further taxonomical and mitogenome comparative studies are essential to resolve the evolutionary relationships of this widely distributed *Rousettus* genus especially within Southeast Asia region.

**Figure 1.**
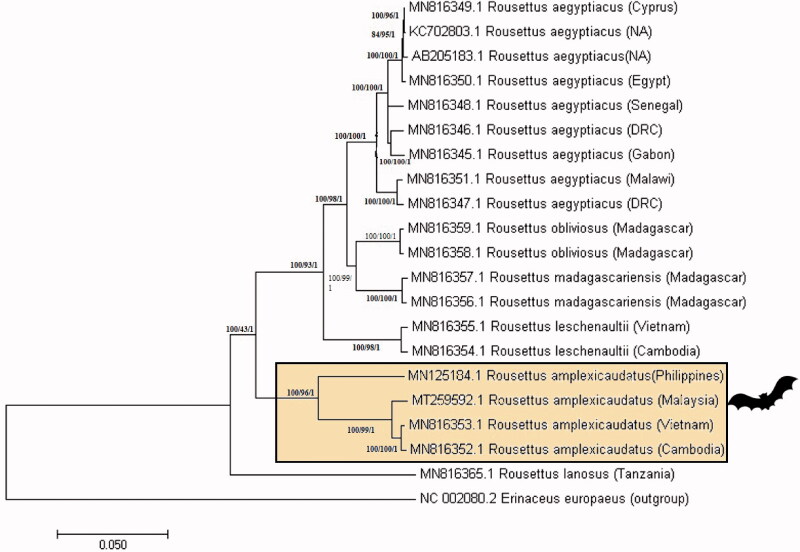
Phylogenetic tree constructed using the whole mitogenome of Malaysian *R. amplexicaudatus* (MT259592) and other *Rousettus* sp. from different countries. The tree was generated from NJ/ML/Bayesian method using hedgehog as an outgroup. Bootstrap values generated from 1000 replicates for NJ/ML/Bayesian analysis. The number at each node indicated the bootstrap probability of NJ/ML/Bayesian analysis. (NA: not available; DRC: Democratic Republic of the Congo).

## Data Availability

The data that support the findings of this study are openly available in the National Center for Biotechnology Information (NCBI) at https://www.ncbi.nlm.nih.gov, accession number MT259592.

## References

[CIT0001] Almeida FC, Giannini NP, Simmons NB. 2016. The evolutionary history of the African fruit bats (Chiroptera: Pteropodidae). *Acta Chiropterologica/Museum and Institute of Zoology*. Pol Acad Sci. 18(1):73–108.

[CIT0002] Chan AAQ, Aziz SA, Clare EL, Coleman JL. 2020. Diet, ecological role and potential ecosystem services of the fruit bat, Cynopterus brachyotis, in a tropical city. Urban Ecosyst. 23(1):1–13.

[CIT0003] Francis C. 2019. Field guide to the mammals of South-east Asia. 2nd ed. London: Bloomsbury Publishing.

[CIT0004] Happold M, Happold DC. 2013. Mammals of Africa: hedgehogs, shrews and bats. London: Bloomsbury Pub.

[CIT0005] Hassanin A, Bonillo C, Tshikung D, Pongombo Shongo C, Pourrut X, Kadjo B, Nakouné E, Tu VT, Prié V, Goodman SM. 2020. Phylogeny of African fruit bats (Chiroptera, Pteropodidae) based on complete mitochondrial genomes. J Zool Syst Evol Res. 39:1.

[CIT0006] Jahari PNS, Abdul Malik NF, Shamsir MS, Gilbert MTP, Mohd Salleh F. 2020. The first complete mitochondrial genome data of Hippocampus kuda originating from Malaysia. Data Brief. 31:105721. 32490085 10.1016/j.dib.2020.105721 PMC7260291

[CIT0007] Mendoza RVD, Fontanilla IKC. 2019. Whole mitochondrial genome of a Geoffroy’s Rousette, *Rousettus amplexicaudatus* (Pteropodidae). Mitochondrial DNA Part B. 4(2):3546–3548. 33366079 10.1080/23802359.2019.1676671 PMC7707315

[CIT0008] Mohd Salleh F, Ramos-Madrigal J, Peñaloza F, Liu S, Mikkel-Holger SS, Riddhi PP, Martins R, Lenz D, Fickel J, Roos C, et al. 2017. An expanded mammal mitogenome dataset from Southeast Asia. Gigascience. 6(8):1–8. 10.1093/gigascience/gix053 PMC5737531 28873965

[CIT0009] Munian K, Azman SM, Ruzman NA, Fauzi NFM, Zakaria AN. 2020. Diversity and composition of volant and non-volant small mammals in northern Selangor State Park and adjacent forest of Peninsular Malaysia. BDJ. 8:e50304. 32317855 10.3897/BDJ.8.e50304 PMC7156494

[CIT0010] Stribna T, Romportl D, Demjanovič J, Vogeler A, Tschapka M, Benda P, Horáček I, Juste J, Goodman SM, Hulva P. 2019. Pan African phylogeography and palaeodistribution of rousettine fruit bats: ecogeographic correlation with Pleistocene climate vegetation cycles. J Biogeogr. 46(10):2336–2349.

[CIT0011] Yoon KB, Kim JY, Park YC. 2016. Characteristics of complete mitogenome of the lesser short-nosed fruit bat *Cynopterus brachyotis* (Chiroptera: Pteropodidae) in Malaysia. Mitochondrial DNA A DNA Mapp Seq Anal. 27(3):2091–2092. 25418628 10.3109/19401736.2014.982571

